# Evaluation of the Hypoglycemic Effects of Flavonoids and Extracts from *Jatropha gossypifolia* L.

**DOI:** 10.3390/molecules20046181

**Published:** 2015-04-09

**Authors:** Sergio Granados, Norman Balcázar, Alis Guillén, Fernando Echeverri

**Affiliations:** 1 Grupo de Genética Molecular y Departamento de Fisiología y Bioquímica, Facultad de Medicina, Universidad de Antioquia, Calle 67 No. 53-10, Medellín 050010, Colombia; E-Mails: micr_clin@hotmail.com (S.G.); norman.balcazar@udea.edu.co (N.B.); alisgui@gmail.com (A.G.); 2 Grupo de Quimica Orgánica de Productos Naturales, Instituto de Química, Universidad de Antioquia, Calle 67 No. 53-10, Medellín 050010, Colombia

**Keywords:** *Jatropha gossypifolia* L., flavanone, flavone, glucose regulation, myotubes, mice

## Abstract

*Jatropha gossypifolia* L. (Euphorbiaceae) is a plant widely used in the treatment of type 2 diabetes mellitus (T2DM), but there are few scientific reports validating its activity in this area. In this work and through a bioguided assay, a crude extract stimulated glucose uptake in C2C12 myotubes up to 30%, thereby reducing insulin resistance induced by fatty acids compared to the basal control. A chromatographic fraction applied intraperitoneally (IP) in mice reduced glucose by 42% in a mouse model of T2DM, after administration of 10 doses during 20 days. A flavanone was purified from this active fraction and its structure was assigned by ^1^H- and ^13^C-NMR (1D and 2D) and MS. This compound retains the previously reported activity, stimulating *in vitro* the glucose uptake in a concentration-dependent manner. This study indicates that *Jatropha gossypifolia* L. extracts enhance glucose uptake in cultured myotubes and adipocytes and also improving glucose tolerance in an *in vivo* model.

## 1. Introduction

Type 2 Diabetes Mellitus (T2DM) is a chronic degenerative disease characterized by alterations in the metabolism of lipids, carbohydrates and proteins [1] and is caused by a decrease in insulin secretion, target-tissue resistance and increased hepatic glucose output. The resulting hyperglycemia is responsible for the increased formation and accumulation of advanced glycation end products, which play an important role in the diabetic complications, such as retinopathy, neuropathy and renal dysfunction [2]. There were an estimated 366 million people with diabetes worldwide in 2011 and at least 4.6 million of them died of this disease.

Therapies currently available for the treatment of the different types of diabetes include insulin and various hypoglycemic agents such as sulphonylureas and biguanides. However, the side effects of these therapies [3] as well as daily intravenous injection in the case of insulin indicate that new and more effective drugs are needed. On the other hand, several compounds from plants used as anti-diabetics have been isolated and identified [4]. Furthermore, since traditional use of plants does not differentiate between types of diabetes treatments based on traditional medicine often do not work properly. Additionally, ethnomedical uses have rarely been validated through laboratory tests of *in vitro* and of *in vivo* models.

Aqueous decoctions of *J. gossypifolia* are used as antidiabetic in Colombia and the Dominican Republic [5]. Besides, in India ethanolic extracts applied in single or multiple doses to a diabetic rats model showed a reduction in glucose levels [6,7]. In spite of that, the compounds responsible for the pharmacological action were not identified.

This current study aimed to analyze the use of *J. gossypifolia* by assessing the activity of the extracts, the chromatographic fractions thereof and the purified substances, in two cell lines involved in the pathology of T2DM (increased glucose uptake) and in an *in vivo* model of T2DM (hypoglycemic activity).

## 2. Results and Discussion

### 2.1. Glucose Uptake in Myotubes and Adipocytes Treated with Extracts of Jatropha gossypifolia L.

**Figure 1:**
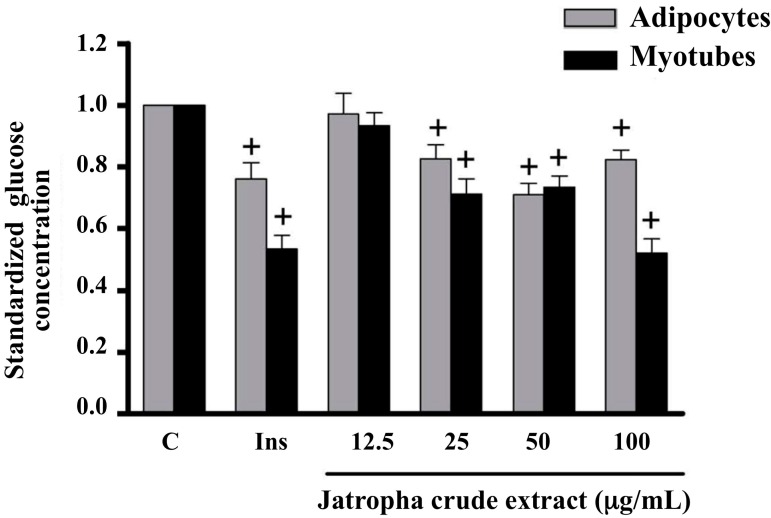
Effect of crude *Jatropha* extract on glucose uptake using cultured C2C12 myotubes and 3T3-L1 adipocytes. The crude extract stimulated glucose uptake in both cell lines. Cells were treated with different concentrations of the extract, and glucose was measured in culture supernatants after 4 h of treatment by the glucose oxidase technique. Bars represent C (control plus DMSO as vehicle), Ins (100 nM insulin), and crude *Jatropha* extract at increasing concentrations. Bar values correspond to the arithmetic mean of glucose concentration for each treatment/Control glucose concentration, *n* = 6. + Indicates *p* < 0.05 for ANOVA and Dunnett’s multiple-range tests. Error bars represent SEM.

C2C12 myotubes and 3T3-L1 adipocytes were incubated with different concentrations (12.5, 25, 50 and 100 µg/mL) of crude *Jatropha* extract for 4 h in glucose uptake medium (5 mM glucose DMEM, 100 µg/mL streptomycin sulfate and 100 Units/mL penicillin, without FBS). Insulin at 100 nM was used as a positive control. The extract showed a concentration-dependent effect, with a significant increase by 148% in glucose uptake from 25 µg/mL to 100 µg/mL in C2C12 myotubes (Figure 1). This percentage was similar to the rate obtained with the presence of 100 nM insulin (46%). In 3T3-L1 adipocytes, the highest uptake (29%) was observed with crude *Jatropha* extract at 50 µg/mL, which was slightly higher than the rate with insulin 100 nM (24%).

### 2.2. Glucose Uptake in Insulin-Resistant Myotubes Treated with Extracts of Jatropha gossypifolia L.

**Figure 2:**
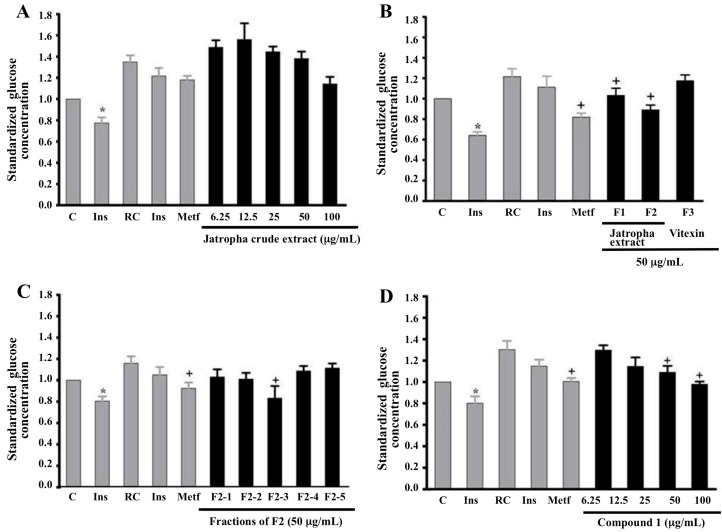
Glucose uptake in C2C12 insulin-resistant myotubes. (**A**) Cells treated with crude *Jatropha* extract; (**B**) Cells treated with diethyl ether F1, ethyl acetate F2 extracts and vitexin F3; (**C**) Cells treated with chromatographic fractions F2-1 to F2-5; (**D**) Cells treated with compound **1**. C = Non-resistant control; Ins = treated with 100 nM insulin; RC = resistant control; Ins treated with 100 nM insulin; Met = treated with 1 mM metformin. The experiments were completed in triplicate. * Indicates *p* < 0.05 for Student’s *t*-test comparison of Control *vs.* Insulin Control. + Indicates *p* < 0.05 for ANOVA and Duncan’s multiple-range tests. Error bars represent SEM.

Compared to the non-resistant control (C), cells treated with sodium palmitate (resistant control), the glucose uptake from basal state (RC) experienced a relevant reduction (16.3%) in glucose uptake (see Figure 2A–D). Similarly, the effect of insulin on glucose uptake was decreased when cells were treated with sodium palmitate. Insulin stimulation of myotubes resulted in a 22% glucose uptake in non-resistant controls (Ins C) compared to 7.4% glucose uptake in resistant controls (Ins RC). These data indicate that palmitate decreased the effect of insulin by 66%, suggesting that these cells are resistant to this hormone. The crude extract of *Jatropha* at 100 µg/mL stimulated glucose uptake by 16% in resistant cells, a much lower rate than that found in non-resistant cells. Metformin, which was used as a positive control, stimulated uptake by 12%. (Figure 2A) Although these findings were not statistically significant, in the present work, by using bio-guided fractionation, we isolate a higher purified material, since crude extract possess a lot of polyphenols, which could disturb many bioassays. Although the target tissue of metformin is the liver, there are several scientific reports indicating significant effects of this drug on skeletal muscle [8].

### 2.3. Effect of Chromatographic Fractions and Pure Compounds on Glucose Uptake in Insulin-Resistant Myotubes

Two extracts obtained by partitioning *Jatropha* crude extract, namely the diethyl ether (F1) and ethyl acetate (F2) fractions, in addition to vitexin (F3), were evaluated. The response to insulin was significantly affected, with the uptake ratio (the arithmetic mean of glucose concentration for each treatment/control glucose concentration) varying from 36% to 8% when cells were treated with palmitate resulting in an 80% of decrease in the effect of insulin. The ethyl acetate extract F2 was the only fraction that showed increased activity, with a glucose uptake ratio in resistant cells 27% higher than that in resistant control cells (RC). These levels were similar to those obtained with non-resistant cells stimulated with insulin (Control) (Figure 2B). In the other hands, vitexin F3, did not show statistical differences compared to the resistant control (RC) (Figure 2B). Moreover, some flavonoids have been reported to be inhibitors of glucose transporters (GLUTs) [9].

Due to the fact that ethyl acetate extract F2 showed high *in vitro* uptake-inducing activity, it was further fractionated by Sephadex LH-20 column chromatography using a mixture of three eluents to afford five subfractions (F2-1, F2-2, F2-3, F2-4 and F2-5). These subfractions were then tested in resistant cells using the same methodology of the previous uptake assays. Fractions F2-1, F2-2, and F2-3 stimulated glucose uptake, the last one having the greatest effect (27%) compared to the resistant control (Figure 2C). Subfractions F2-4 and F2-5 showed high polarity and no effects on activity.

The *J. gossypifolia* crude extract did not significantly affect cell viability, as its IC_50_ was higher than 100 µg/mL. Ethyl acetate extract F2 and vitexin exhibited a IC_50_ higher than 200 μg/mL while this value in fraction F1 was lower than 57 µg/mL (Table 1).

**Table 1:** IC_50_ (cell viability) of the extracts. SD = standard deviation. Each experiment was completed in triplicate.

Plant Material	IC_50_ (µg/mL) C2C12	SD	IC_50_ (µg/mL) HepG2	SD
Crude *Jatropha* extract	135	4.6	108	14
Diethyl ether (F1)	57	12.7	70	9.7
Ethyl acetate (F2)	>200	6.9	>200	5.6
Vitexin (F3)	>200	4.3	>200	8.3

### 2.4. Effect of Compound **1** on Glucose Uptake

In the active chromatographically separated fraction, three substances were detected, from which the major product **1** was isolated and identified. This product stimulated glucose uptake in insulin-resistant cells in a concentration-dependent manner, up to 27% at 50 μg/mL compared to the uptake of insulin-resistant cells (RC). (Figure 2D) The two additional compounds were not tested, as they could not be structurally identified due to a low concentration of the active fraction.

### 2.5. Structure of Compound **1** with Hypoglycemic Effects

**Figure 3:**
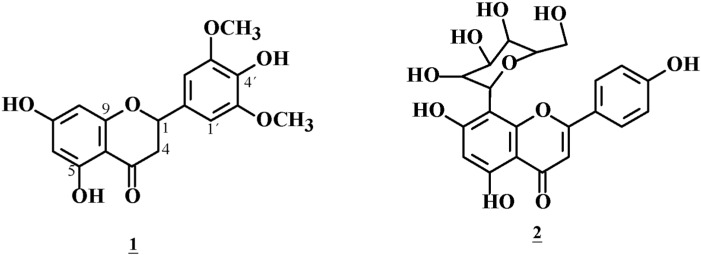
Structure of the hypoglycemic flavanone **1** and vitexin **2**, a suppressor of glucose uptake.

The bioguided isolation of the crude methanol extract by Sephadex LH-20 and silica-gel chromatography, yielded several chromatographic fractions that increase glucose uptake in myotubes and adipocytes. A pure compound **1** was obtained after a preparative TLC of the active fraction, whose structure was elucidated by MS and NMR (COSY, HMQC, APT and HMBC) (Figure 3) and identified as 5,7,4'-trihydroxy-3',5'-dimethoxyflavanone. The following characteristic flavanone signals were detected: 2.81 ppm (dt, *J* = 14.0 and 4.0, H3eq), 3.14 (dd, H = 14.0 and 12.0, H3ax), 3.97 (s, 6H, C3'-OCH_3_ and C5'-OCH_3_), and 5.35 (dd, *J* = 12.0 and 4.0, H-2). In addition, there were signals for a tetra- substituted B-ring, for which there was a singlet integrating for two signals, one of them at 6.72 ppm, assignable to H2' and H6', and the second one at 6.05 ppm, assignable to H6 and H8. Similarly, the carbon atoms were assigned as follows: 43.61 ppm (C3), 55.75 (OCH_3_), 56.43 (OCH_3_), 79.65 (C1), 94.35 (C6), 95.21 (C8), 103.19 (C2', C6', C10), 129.43 (C4'), 135.23 (C1'), 147.26 (C3' and C5'), 164.17 (C9), 168.04 (C10), 177.70 (C7), and 195.96 (C4). This compound was reported in 2012, but its hypoglycemic activity was not known [10]; in addition other flavonoids have been reported as hypoglycemic in TD2 [11].

### 2.6. Structure of the Glucose Uptake Suppressor

The structure of the yellow precipitate F3 extract with a negative effect on glucose uptake was assigned using the same spectroscopic methodology previously described. This compound corresponded to the flavonoid vitexin (Figure 3), which is very common in the plant kingdom [12].

### 2.7. Intraperitoneal Glucose Tolerance Test (IPGTT)

To determine glucose tolerance of diabetic mice among those animals treated with ethyl acetate extract F2, intraperitoneal glucose tolerance tests were performed. This route of administration was selected on the basis of a best and fast bioavailability. The results were analyzed as total area under the curve (AUC) from time 0 to 120 min. The results indicate a decrease in glucose tolerance in diabetic mice (HFD + STZ) compared to controls (fed with standard diet) because the AUC is greater in the diabetic group (data not shown). 

**Figure 4:**
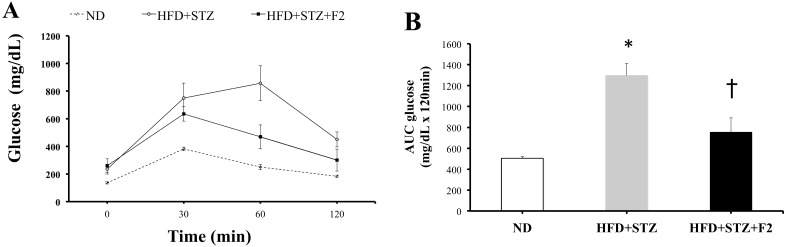
(**A**) IPGTT after 10 IP treatment doses; (**B**) Area under the curve (AUC) values. Times 1, 2, 3 and 4 correspond to 0 min, 30 min, 60 min and 120 min, respectively. ND = normal diet group, HFD+STZ = diabetic control group, and HFD + STZ + ethyl acetate extract F2 = diabetic group given 10 IP doses of ethyl acetate extract F2). * Indicates *p* < 0.05 for Student’s *t*-test comparison of ND *vs.* HFD+STZ. † Indicates *p* < 0.05 for Student’s *t*-test comparison of HFD + STZ *vs.* HFD + STZ + F2. Error bars represent SEM.

After 10 intraperitoneal doses administered at a dose of 20 mg/kg, in alternate day dosing to avoid stress (20 days in total), mice continued to show fasting hyperglycemia (Figure 4A), although the AUC values indicated that these mice maintained 42% lower glucose levels than the HFD + STZ diabetic mice, suggesting improved glucose tolerance in the group of diabetic mice that received ethyl acetate extract F2 (Figure 4B). 

T2DM is a chronic degenerative disease affecting the world’s population and is a major cause of morbidity, disability and mortality. It is considered one of the most common major global non-communicable diseases and is the fourth or fifth leading cause of death in both developed and developing countries and its incidence is increasing. It is expected that over the next 30 years, there could be an increase of more than 100% in diagnoses, because of a higher prevalence of overweight and obese children [13]. 

The results of this study show that the crude extract of *Jatropha* significantly stimulates *in vitro* glucose uptake in two cell models (C2C12 myotubes and 3T3-L1 adipocytes) with an exposure time of 4 h (Figure 2), although this effect was higher in myotubes than in adipocytes. The first is a feasible model to evaluate glucose uptake if the systemic role of muscle tissue in maintaining blood glucose homeostasis is considered. Besides, the C2C12 cell line has been well established in the study of events related to both processes, signaling and responses to insulin; however, the uptake ratio changes depending on the quality of cultures, the size of myotubes structures and exposure time. In the current study, it was found that uptake ratio in this model were approximately 50% of the basal control (Figure 1 and Figure 2B); this rate is similar to previous reports [14]. 

In this work, insulin resistance was also induced in differentiated myotubes to validate the hypoglycemic effect of the plant extracts in an *in vitro* model that is more similar to the conditions present in insulin-resistant individuals. Several studies have reported that palmitate may induce insulin resistance in myotubes and significantly affect glucose uptake [15]. The results indicated a loss of insulin sensitivity of up to 80%, which caused a decrease in glucose uptake ratio by as much as 24% (Figure 2B), while metformin (1.0 mM), which inhibits hepatic glucose production and stimulates skeletal muscle uptake, increased glucose uptake in our study by 20% with respect to the resistant myotubes control. 

The extracts and chromatographic fractions analyzed also showed no cytotoxicity according to a MTT assay. (Table 1) Similarly, palmitate at 0.75 mM did not significantly affect cell viability within 72 h of exposure; viability was reduced by only 7% (data not shown).

It has been previously reported that other flavonoids have had this effect on different cell lines due to the decrease in the translocation of glucose transporter (GLUT) [16] and blockade of protein kinase phosphorylation. This action may explain why a possible antitumor potential effect has also been attributed to flavonoids [17]. However, the molecular mechanisms are still not well established, perhaps due to the numerous and diverse biological effects that have been described in different cells and tissues [18]. 

The chromatographic fraction F2 was administered in the mouse model to verify its effects *in vivo*. A model of T2DM, generated through a high fat diet and low doses of STZ, resembles the gradual progression of obesity with insulin resistance, compensatory relief from this resistance, and dysfunction and/or progressive death of pancreatic-cells, reducing insulin secretion. [19] Although mice treated with ethyl acetate extract F2 failed to normalize glucose fasting levels (time 0), there was an improvement in glucose tolerance after treatment, with approximately a 42% reduction (Figure 2D). In this manner, glucose uptake in muscle and adipocytes may be responsible for the reduction seen *in vivo*. 

Although *J. gossypifolia* L. has been used in several countries like a hypoglycemic, but this is the first time the compound responsible for this activity has been isolated and identified. Using bioguided assays, the major component of the active chromatographic fraction was purified, and its structure was elucidated using spectroscopic methods. The structure of this fraction corresponds to a flavanone, which stimulated glucose uptake in C2C12 insulin-resistant myotubes by 27% compared to the resistant control (RC) and displayed an effect similar to the positive control, metformin (Figure 2D). Recently, a report has been published on secondary metabolites of the genus *Jatropha* [20]; surprisingly, the active molecule is a flavanone, which has not been previously reported in this genus, where flavones are the most characteristic flavonoids. Natural compounds are an attractive alternative to synthetic drugs, as they can also serve to support current treatments, either as a developed drug or phytodrug. Between 2001 and 2005, four new drugs derived from natural products were introduced for the treatment of T2DM and dyslipidemia [21].

## 3. Experimental Section 

### 3.1. General Procedures

All solvents used for extraction and fractionations: methanol, ethanol, ethyl acetate, *n*-hexane and dichloromethane were previously distilled from commercial sources. Thin layer chromatographies were running in aluminum-backed F_254_ silica gel chromatoplates (Merck, Darmstadt, Germany). Column chromatographies were performed with Sephadex LH-20 (Sigma, St Louis, MO, USA.) and glass columns (60 × 7 cm) filled with silica gel 60H (Merck) as well as with glass columns (40 × 3 cm). ^1^H-NMR), ^13^C-NMR and two-dimensional spectra were obtained in a Bruker AMX 300 spectrometer (Bruker Bio-Spin GmbH, Rheinstetten, Germany) operating at 300 MHz for ^1^H and 75.0 for ^13^C using CDCl_3_ or DMSO d_6_. Shifts are reported in δ units (ppm) and coupling constants (*J*) in Hz. PBS 1× (Gibco, Carlsbad, CA, USA), sodium bicarbonate, sodium palmitate (Sigma) and 100 IU/mL insulin (Humulin, Eli Lilly, Indianapolis, IN, USA) were used. TLC plates were Merck, 0.25 mm, and revealed spraying with H_2_SO4-acetic acid mixture and then heated at 100 °C.

A glucose oxidase-peroxidase kit (BioSystems, Bogotá DC, Colombia) was used for glucose measurement using a Varioskan Flash spectrophotometer (Thermo, Waltham, MA, USA) at 500 nm. For the methyl thiazole tetrazolium (MTT, Amresco, Solon, OH, USA) assay, the same spectrophotometer was used at 570 nm. Glycaemia in mice was measured with the One Touch Ultra Mini Glucometer from Johnson & Johnson (New Brunswick, NJ, USA).

### 3.2. Preparation of the Extract and Chromatographic Fractionation

*Jatropha gossypifolia* L. leaves were collected in Valledupar (Colombia) in 2011. A specimen was deposited in the Herbarium of the University of Antioquia with #178509. The dried leaves (2 kg) were extracted with 4 L of 96% methanol at room temperature during 24 h and the extract was filtered and concentrated to dryness at 38 °C under reduced pressure to afford 120 g of methanol extract. This extract was redissolved in 250 mL of a water/methanol mixture (1:1, v/v) and then partitioned with 400 mL (4 × 100 mL) of ethyl ether and subsequently with 400 mL (4 × 100 mL) of ethyl acetate, to afford fractions F1 (43.4 g) and F2 (32.0 g), respectively. A precipitate was obtained from the last extract, which was washed thoroughly with methanol, yielding 2.5 g of a yellow solid (F3).

Afterward, 8.4 g of the ethyl acetate extract F2 were fractionated on a Sephadex LH-20 column using the mixture petroleum ether/dichloromethane/methanol 2:1:1 (v/v) as eluent, and monitoring by silica gel TLC in hexane/ethyl acetate 1:1 (v/v); five fractions were collected (F2-1 to F2-5), which were analyzed for the *in vitro* glucose uptake. Active fraction F2-3 (453 mg) was separated on a silica gel column, eluted with a petroleum ether/ethyl acetate 5:1 (v/v) mixture, which increased in polarity to finish with pure ethyl acetate; a total of 15 fractions were collected. 

From this new column, the *in vitro* active fractions 11 and 12 (F2-3-11 and F2-3-12) showed the presence of three compounds by TLC in hexane/ethyl acetate (1:1, v/v), which were separated by preparative chromatography in the same system (three runs). In this way, 20 mg of the active substance **1** with hypoglycemic activity were obtained. The other two substances could not be identified due to low purity, low yield and complexity of the NMR spectra. After several washing with methanol, the yellow compound F3 shown to be pure by thin-layer chromatography and structural analysis was elucidated by NMR. 

### 3.3. Cell Cultures

The C2C12 mouse muscle cells were purchased from ATCC (Manassas, VA, USA, CRL-1772); 3T3-L1 adipocytes were kindly donated by Sergio Acín from the Department of Biochemistry and Molecular Biology of the Aragon Institute of Health Sciences at the University of Zaragoza (Spain). Cells were cultured and maintained at 37 °C and 5% CO_2_ in DMEM culture medium with 10% fetal bovine serum (FBS), 2 mM glutamine, penicillin and 1% streptomycin (Sigma). When the cells reached a confluence between 80% and 90%, they were differentiated into myotubes and adipocytes.

C2C12 cells were differentiated into myotubes using low glucose (5.5 mM) DMEM medium supplemented with 5% horse serum (HS). [22] For the glucose utilization experiment, the cells were incubated in the presence and absence of insulin and in the presence of different concentrations of the extracts and/or fractions to be evaluated. After 4 ho of incubation, 500 μL of supernatant were collected, and glucose concentration was measured using the glucose oxidase/peroxidase technique with a commercial kit (BioSystems). To calculate glucose utilization, the remaining glucose in the culture medium after incubation with controls and extracts or fractions was subtracted from the initial amount of glucose (5.5 mM).

3T3-L1 fibroblasts were differentiated into adipocytes with 1 μM insulin, 0.5 mM IBMX and 0.25 μM dexamethasone for 3–5 days. The medium was removed, and the cells were left in only DMEM and 1 μM insulin for 5–7 days. The medium was changed every 2 days until the eighth day of differentiation [23]. One glucose uptake assay was performed as described above for the C2C12 cells.

To generate insulin-resistant cells, 4-day differentiated myotubes were pre-incubated for 2 h in DMEM medium with 5.5 mM glucose without fetal bovine serum and supplemented with 1% BSA; these myotubes were then incubated for 18 h in DMEM medium with 5.5 mM glucose without fetal bovine serum, 1% BSA, 0.75 mM palmitate and 200 mM insulin [24]. Subsequently, DMEM uptake medium was added, supplemented with 5.5 mM glucose and various chromatographic fractions or pure substances, and the cells were cultured for an additional 4 h. One hundred mg of commercial metformin (Laboratorio Memphis, Bogotá DC, Colombia) were pulverized, dissolved in cultured media and used as positive control. Finally, the supernatants were collected, and glucose concentration was measured as described above. 

To evaluated cell viability, HepG2 (ATCC HB-8065) and C2C12 cells were seeded on 96 multi-well plates at 2.5 × 10^4^ cells/well and cultured for 24 h. The cells were washed with DMEM once, and then incubated with different concentration of extracts or fractions (200, 100, 50, 25, 12, 6, 3 and 1.5 μg/mL) in DMEM for 72 h. Subsequently, the medium was removed, cells were washed once, and MTT 5 mg/mL in DMEM were added to each well and incubated for 4 h. The MTT medium was removed and 200 μL of DMSO were added to dissolve the formazan formed. The optical densities (OD) at 570 nm were measured using a spectrophotometer [25]. The IC_50_ was determined by constructing a dose-response curve and examining the effect of different concentrations of extracts or fractions, by linear regression analysis calculated using the Probit method.

### 3.4. Glucose Tolerance Test in the Mouse Model of T2DM

C57BL6/J male mice (Charles Rivers Laboratories, Wilmington, MA, USA) over 4 weeks old were used in this study. Mice were housed at 22 ± 2 °C with a 12:12 h light dark cycle with free access to food and water for 8 weeks. Mice were randomly divided into three groups for this study. A control group (*n* = 10) was fed a normal diet (ND, 14% fat/54% carbohydrates/32% protein). A HFD + STZ group (*n* = 10) and an HFD + STZ + ethyl acetate extract F2 group (*n* = 10) were fed a high-fat diet (HFD, 42% fat/42% carbohydrates/15% proteins) and received 3 doses of streptozotocin (STZ) at a low concentration (25 mg/kg) at the same time each day every day, after 4-h fast. The HFD + STZ + F2 group received 10 intraperitoneal doses, a dose every two days; of ethyl acetate extract F2, 20-mg/kg. All protocols were approved by the Ethics Committee for Animal Research at the University of Antioquia (Protocol number 65). Before and after treatment with ethyl acetate F2, an intraperitoneal glucose tolerance test (IPGTT) was performed on the mice by administering a glucose load of 2.0 g/kg body weight. Glucose levels were measured at 0, 30, 60 and 120 min using a GlucoQuick Glucometer (Procaps, Barranquilla, Colombia). The zero time was measured just before glucose injection.

### 3.5. Statistical Analysis

Results are expressed as means ± s.e.m. Statistical analysis was performed using the ANOVA and Dunnett’s multiple-range tests. Student’s *t*-tests were used to compute individual pairwise comparisons of least square means. The trapezoidal rule was used to determine the area under the curve (AUC). Differences were considered to be significant at *p* < 0.05. All analyses were performed with Prism 4 (GraphPad software, Inc., La Jolla, CA, USA) statistical software.

## 4. Conclusions

The findings of this study are in agreement with the traditional uses of the leaves of *Jatropha gossypifolia* L. as a hypoglycemic agent in some regions of Colombia and other countries. One of the substances responsible for the effects of *J. gossypifolia* is a flavanone that significantly stimulated glucose uptake in C2C12 myotubes under insulin-resistance conditions induced by palmitate. This flavanone represents a new type of compound from which new and more effective drugs could be designed for the treatment of T2DM particularly considering that metformin, a common drug used to control T2DM, has unpleasant side effects and its hypoglycemic effect is typically difficult to maintain consistently. 

*In vivo*, using a mouse model of T2DM, the flavanone-containing fraction, as the major product, significantly reduced the AUC of glucose tolerance by 32% but showed neither a reduction in fasting blood glucose levels nor a reduction to normal levels after IPGTT, although the dose administered was very low. Other trials with pure flavanone are required to adjust the therapeutic regimen.

## Supplementary Materials

Supplementary materials can be accessed at: http://www.mdpi.com/1420-3049/20/04/6181/s1.
