# Supplementary material for: Evaluation of the Hypoglycemic Effects of Flavonoids and Extracts from Jatropha gossypifolia L
Source: Molecules. 2015 Apr 9;20(4):6181–93. doi: 10.3390/molecules20046181 (PMC6272771; doi:10.3390/molecules20046181)
Supplement: Supplementary file 1 [file molecules-20-06181-s001.pdf]

Supplementary Materials

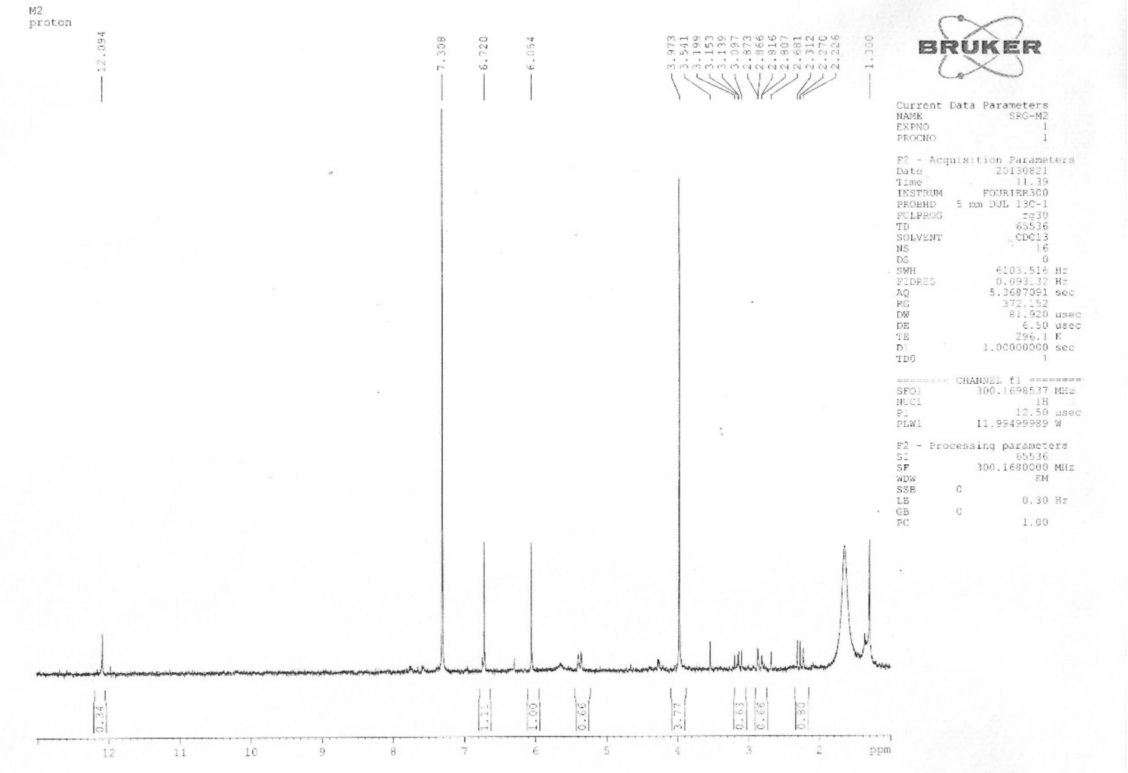

Figure S1. ^{1}H-NMR of compound 1.

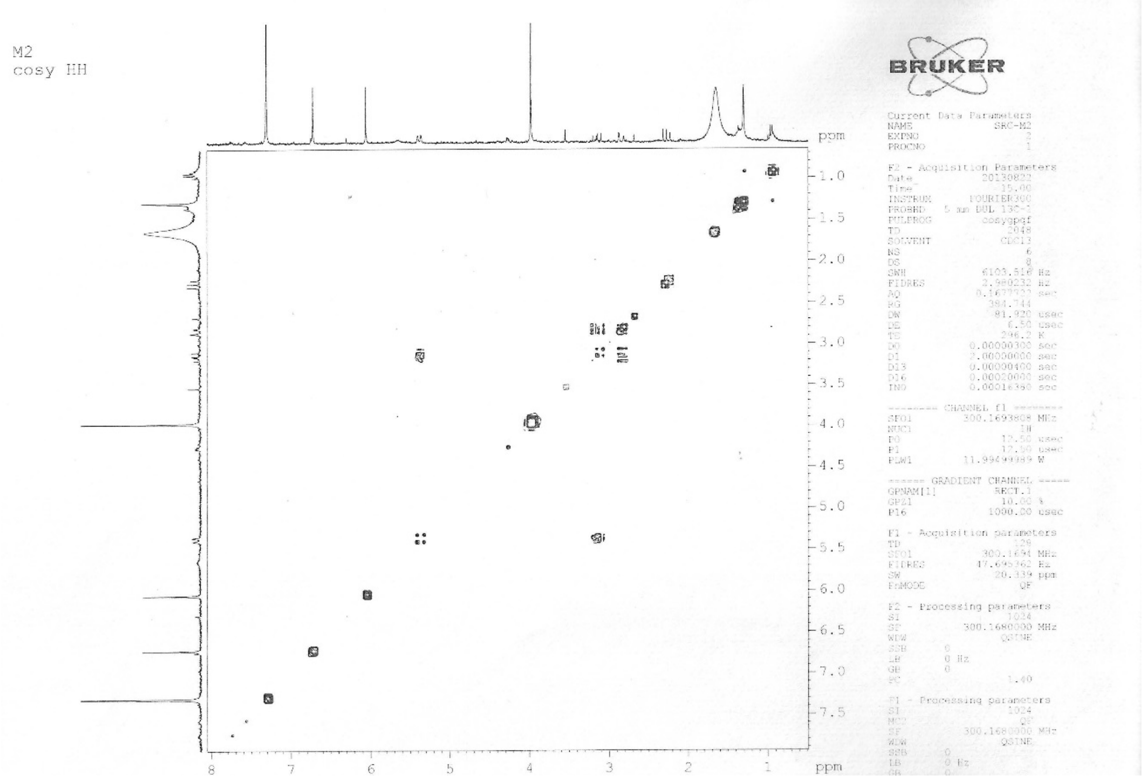

Figure S2. ^{1}H-^{1}H COSY of compound 1.

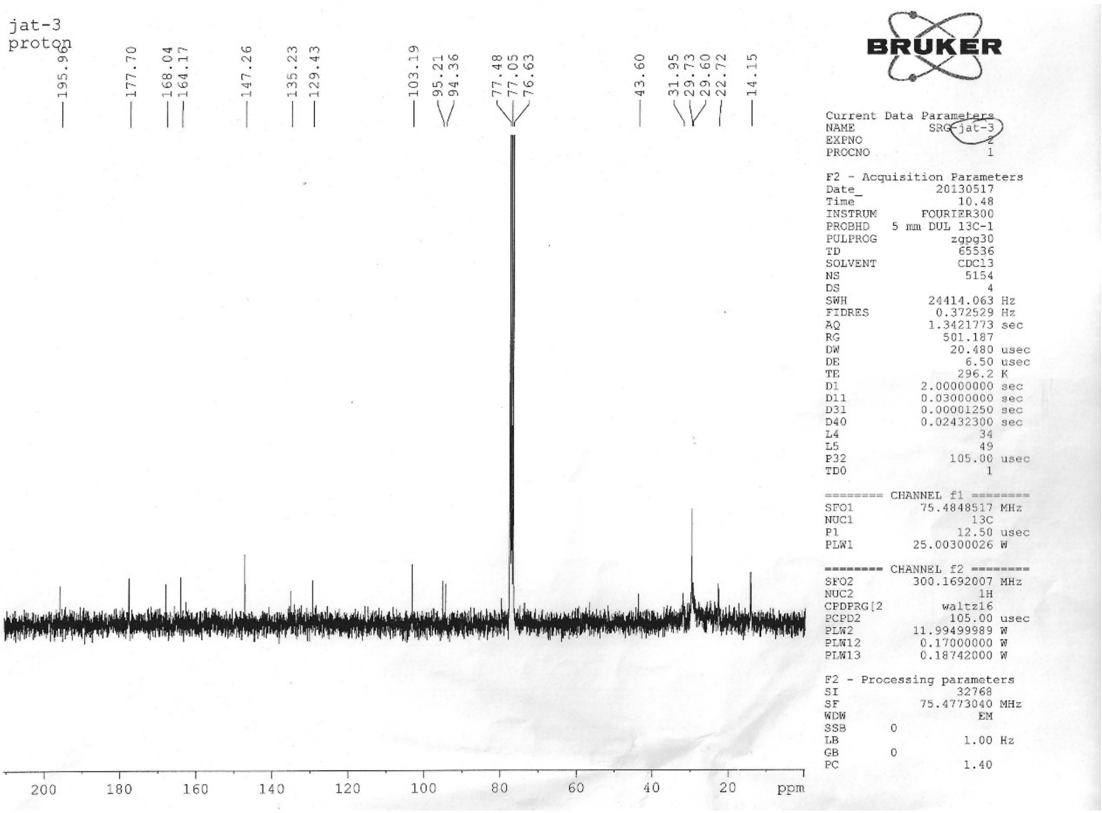

Figure S3. ^{13}C-NMR of compound 1.

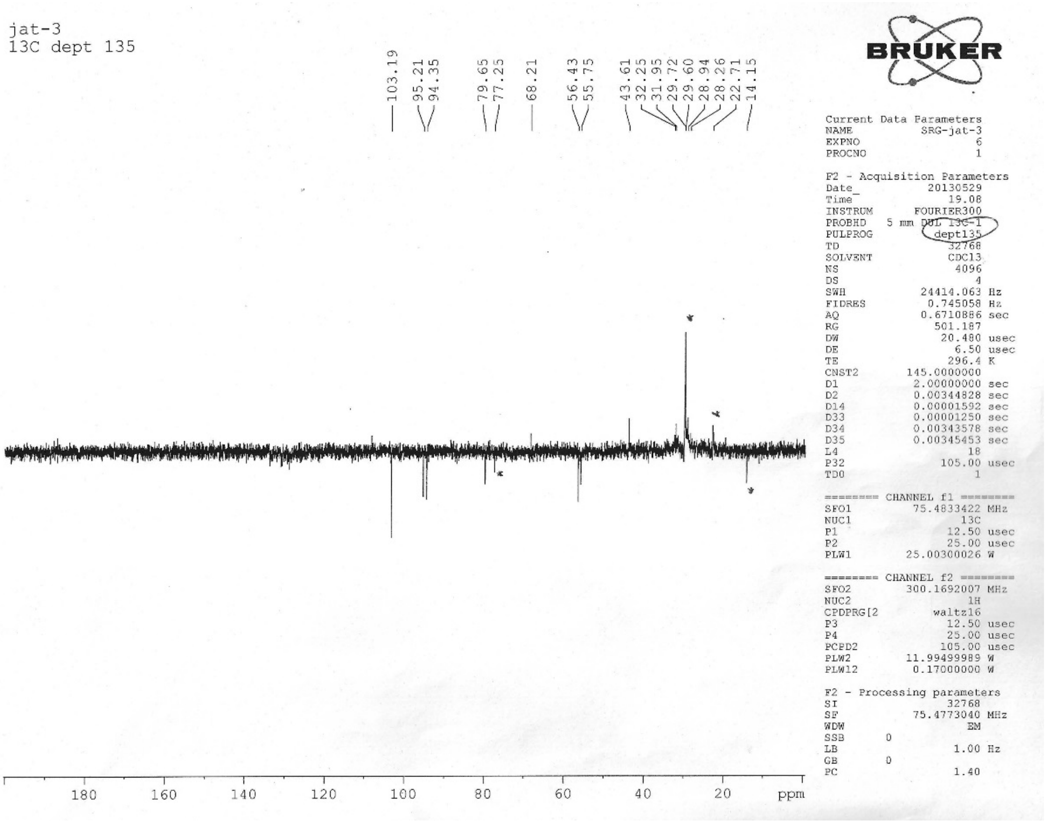

Figure S4. Dept 135 of compound 1.

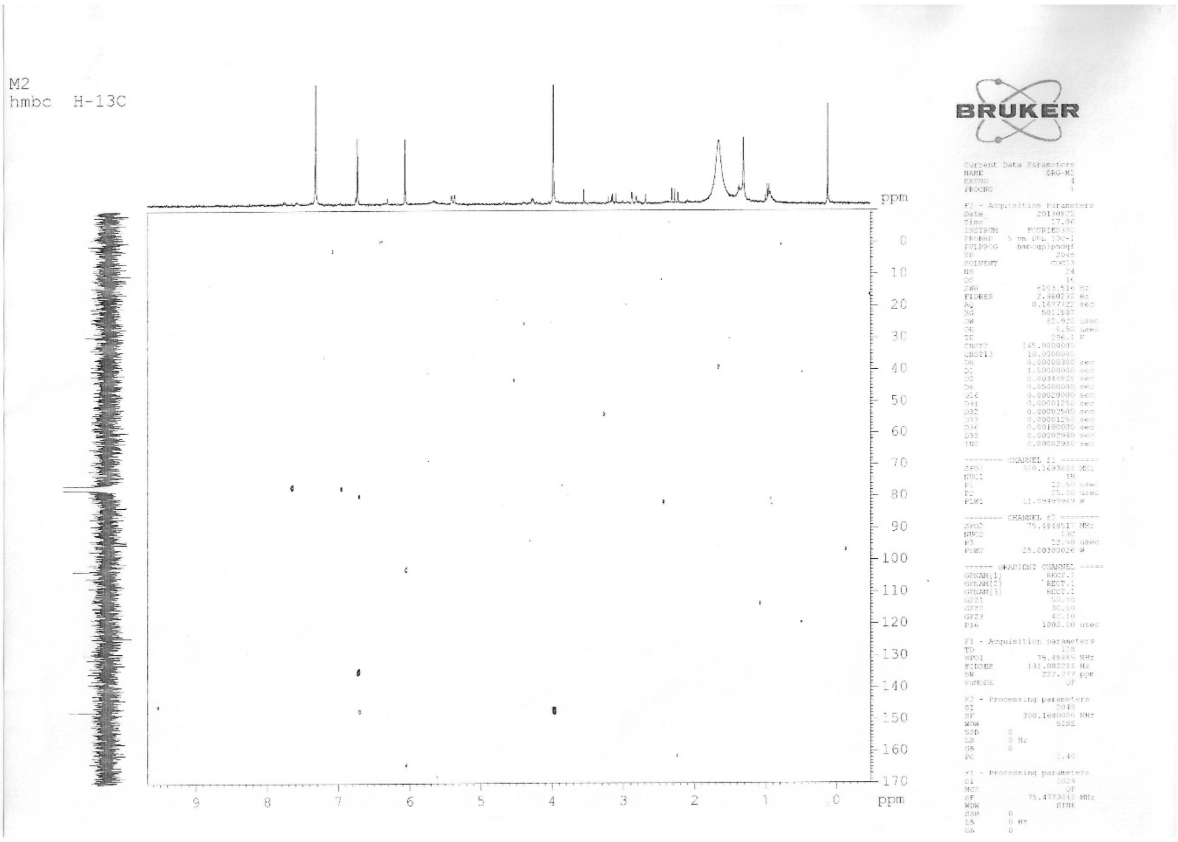

Figure S5. HMBC of compound 1.
